# Supplementary material for: Depletion of Endothelial-Derived 2-AG Reduces Blood-Endothelial Barrier Integrity via Alteration of VE-Cadherin and the Phospho-Proteome
Source: Int J Mol Sci. 2023 Dec 30;25(1):531. doi: 10.3390/ijms25010531 (PMC10778805; doi:10.3390/ijms25010531)
Supplement: Supplementary file 1 [file ijms-25-00531-s001.zip › ijms-2794446-supplementary.pptx]

## Slide 1
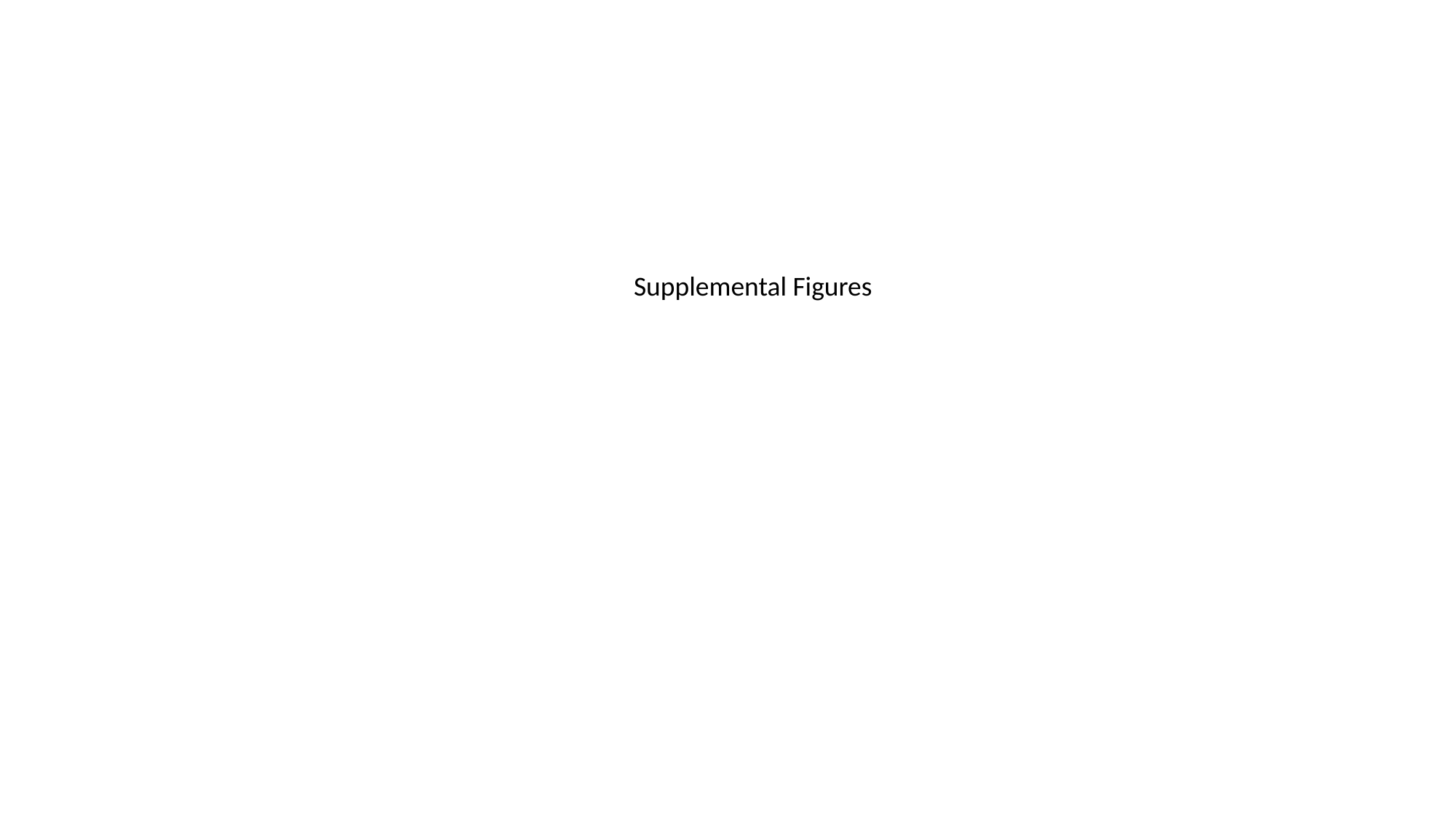

Supplemental Figures

## Slide 2
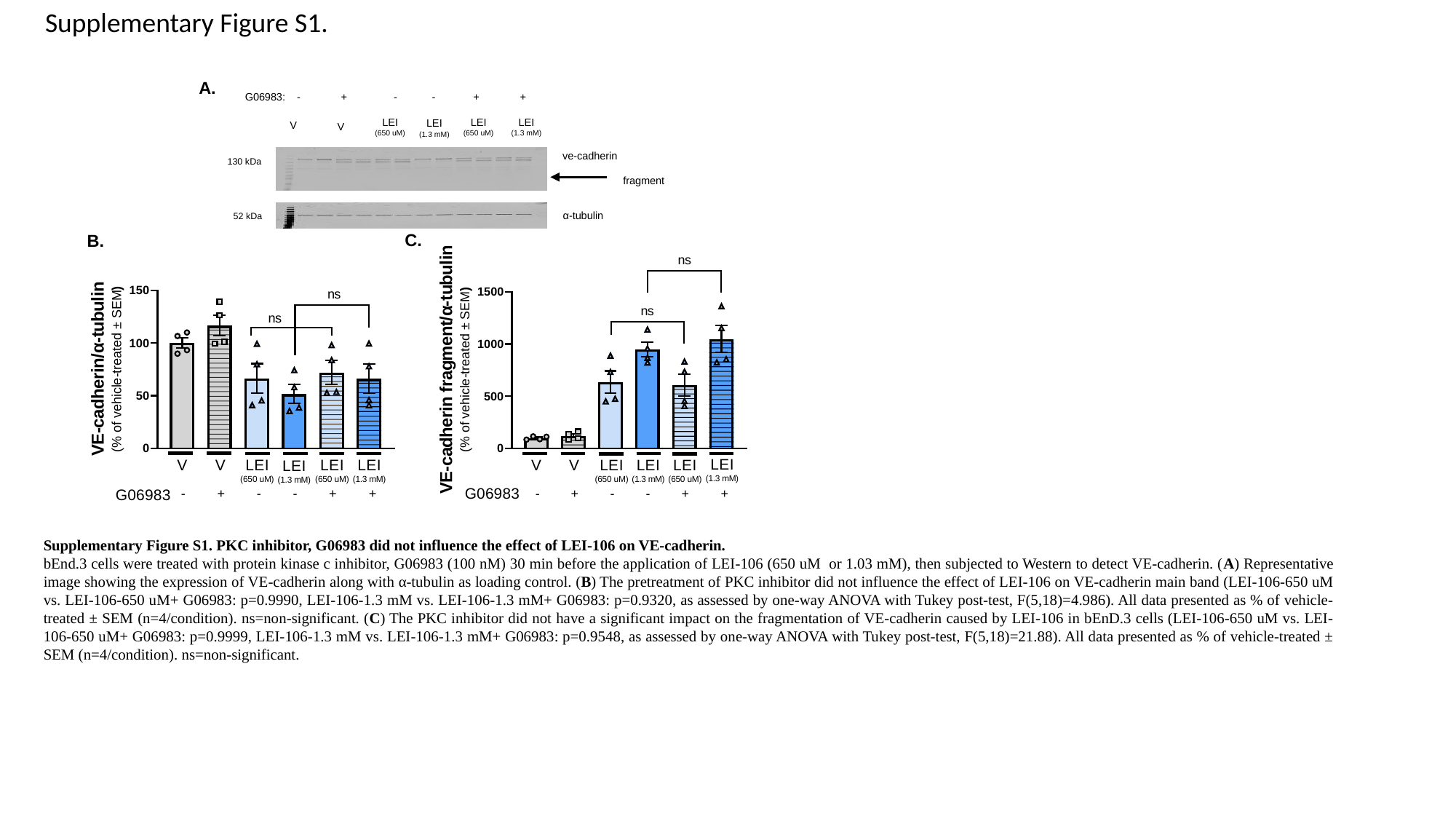

Supplementary Figure S1.
A.
G06983: - + - - + +
LEI
(1.3 mM)
LEI
(650 uM)
LEI
(650 uM)
LEI
(1.3 mM)
V
V
ve-cadherin
130 kDa
fragment
α-tubulin
52 kDa
C.
B.
Supplementary Figure S1. PKC inhibitor, G06983 did not influence the effect of LEI-106 on VE-cadherin.
bEnd.3 cells were treated with protein kinase c inhibitor, G06983 (100 nM) 30 min before the application of LEI-106 (650 uM or 1.03 mM), then subjected to Western to detect VE-cadherin. (A) Representative image showing the expression of VE-cadherin along with α-tubulin as loading control. (B) The pretreatment of PKC inhibitor did not influence the effect of LEI-106 on VE-cadherin main band (LEI-106-650 uM vs. LEI-106-650 uM+ G06983: p=0.9990, LEI-106-1.3 mM vs. LEI-106-1.3 mM+ G06983: p=0.9320, as assessed by one-way ANOVA with Tukey post-test, F(5,18)=4.986). All data presented as % of vehicle-treated ± SEM (n=4/condition). ns=non-significant. (C) The PKC inhibitor did not have a significant impact on the fragmentation of VE-cadherin caused by LEI-106 in bEnD.3 cells (LEI-106-650 uM vs. LEI-106-650 uM+ G06983: p=0.9999, LEI-106-1.3 mM vs. LEI-106-1.3 mM+ G06983: p=0.9548, as assessed by one-way ANOVA with Tukey post-test, F(5,18)=21.88). All data presented as % of vehicle-treated ± SEM (n=4/condition). ns=non-significant.

## Slide 3
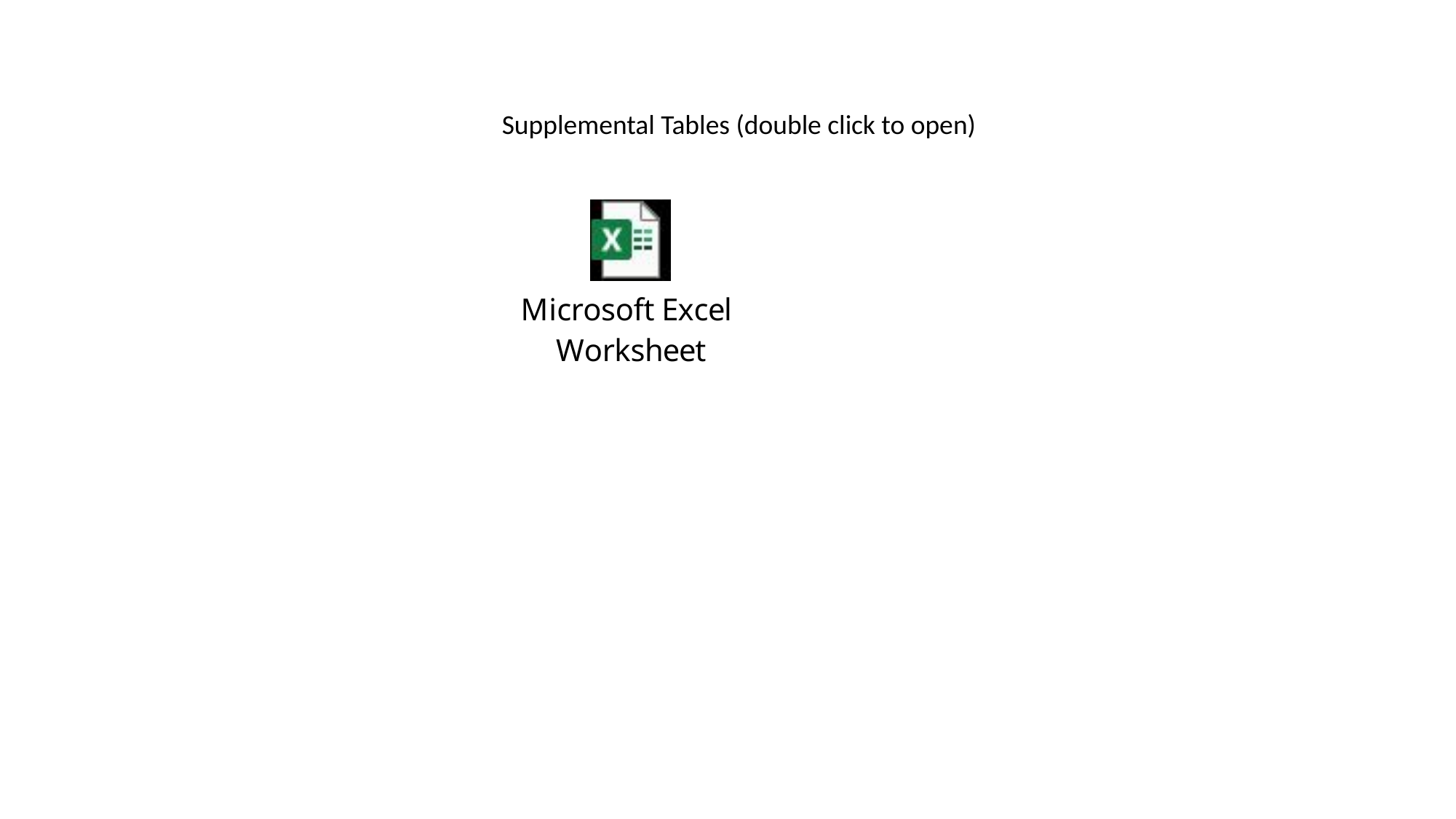

Supplemental Tables (double click to open)

## Slide 4
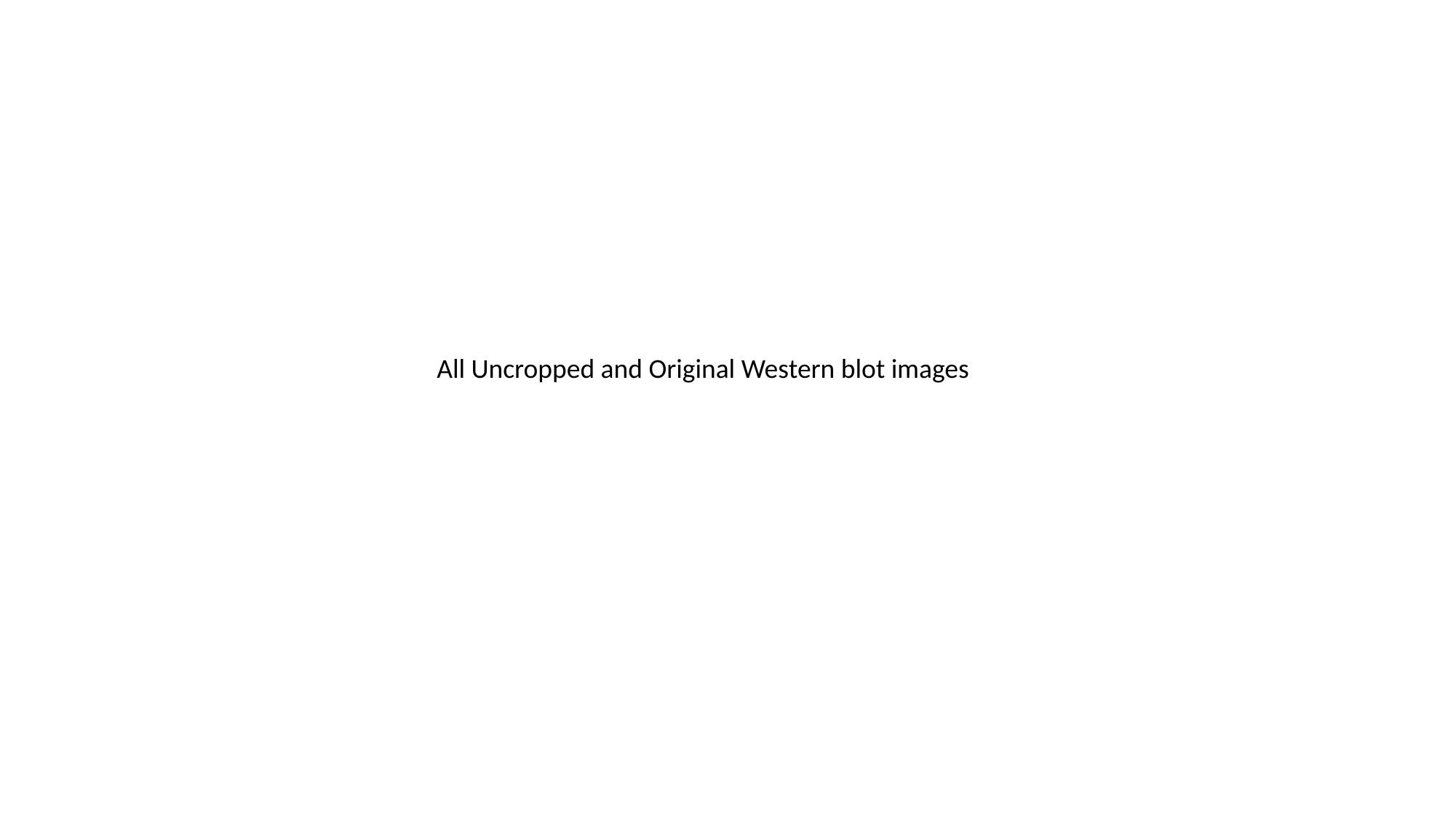

All Uncropped and Original Western blot images

## Slide 5
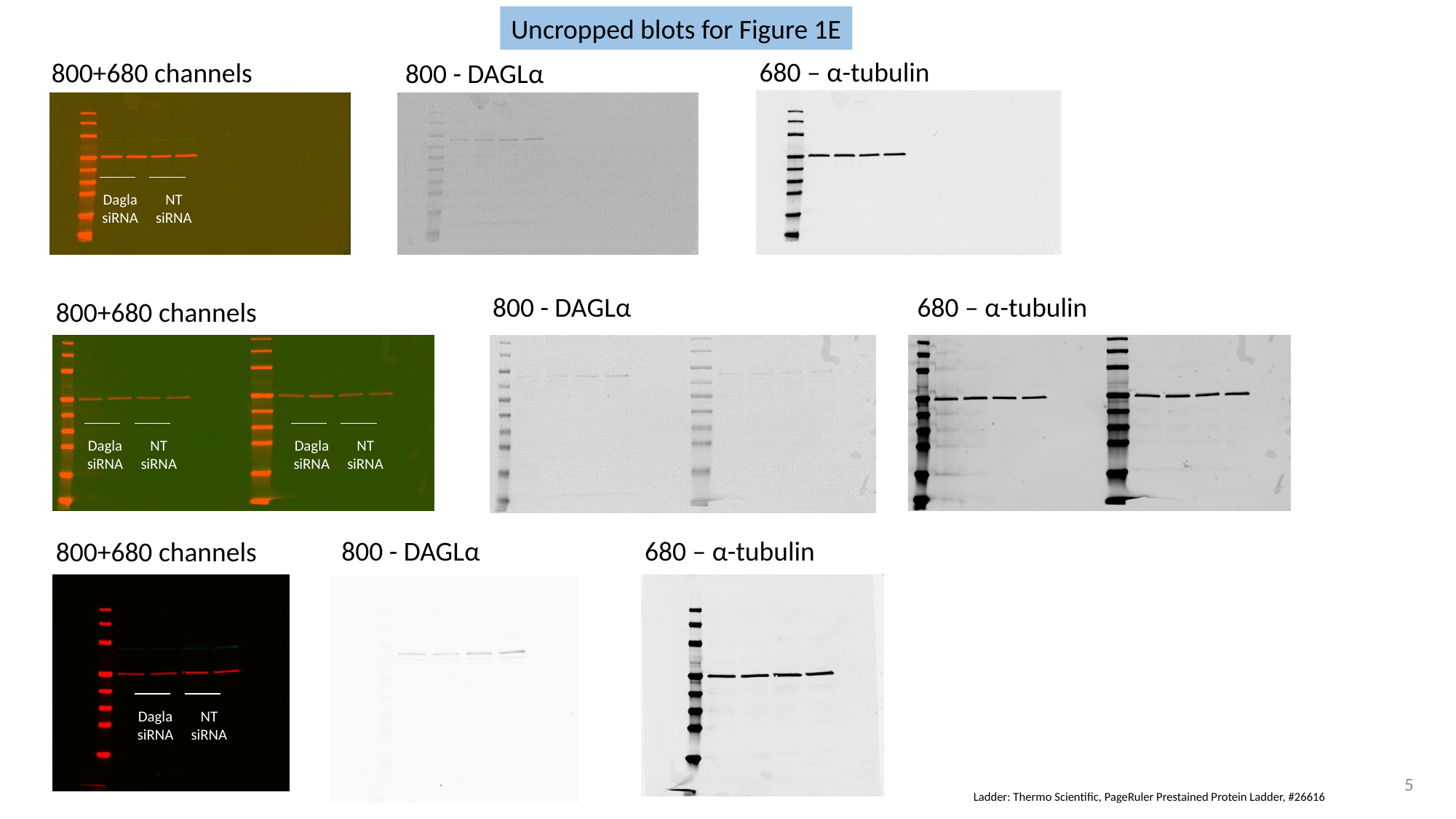

Uncropped blots for Figure 1E
680 – α-tubulin
800+680 channels
800 - DAGLα
Dagla
siRNA
NT
siRNA
800 - DAGLα
680 – α-tubulin
800+680 channels
Dagla
siRNA
NT
siRNA
Dagla
siRNA
NT
siRNA
800 - DAGLα
680 – α-tubulin
800+680 channels
Dagla
siRNA
NT
siRNA
5
Ladder: Thermo Scientific, PageRuler Prestained Protein Ladder, #26616

## Slide 6
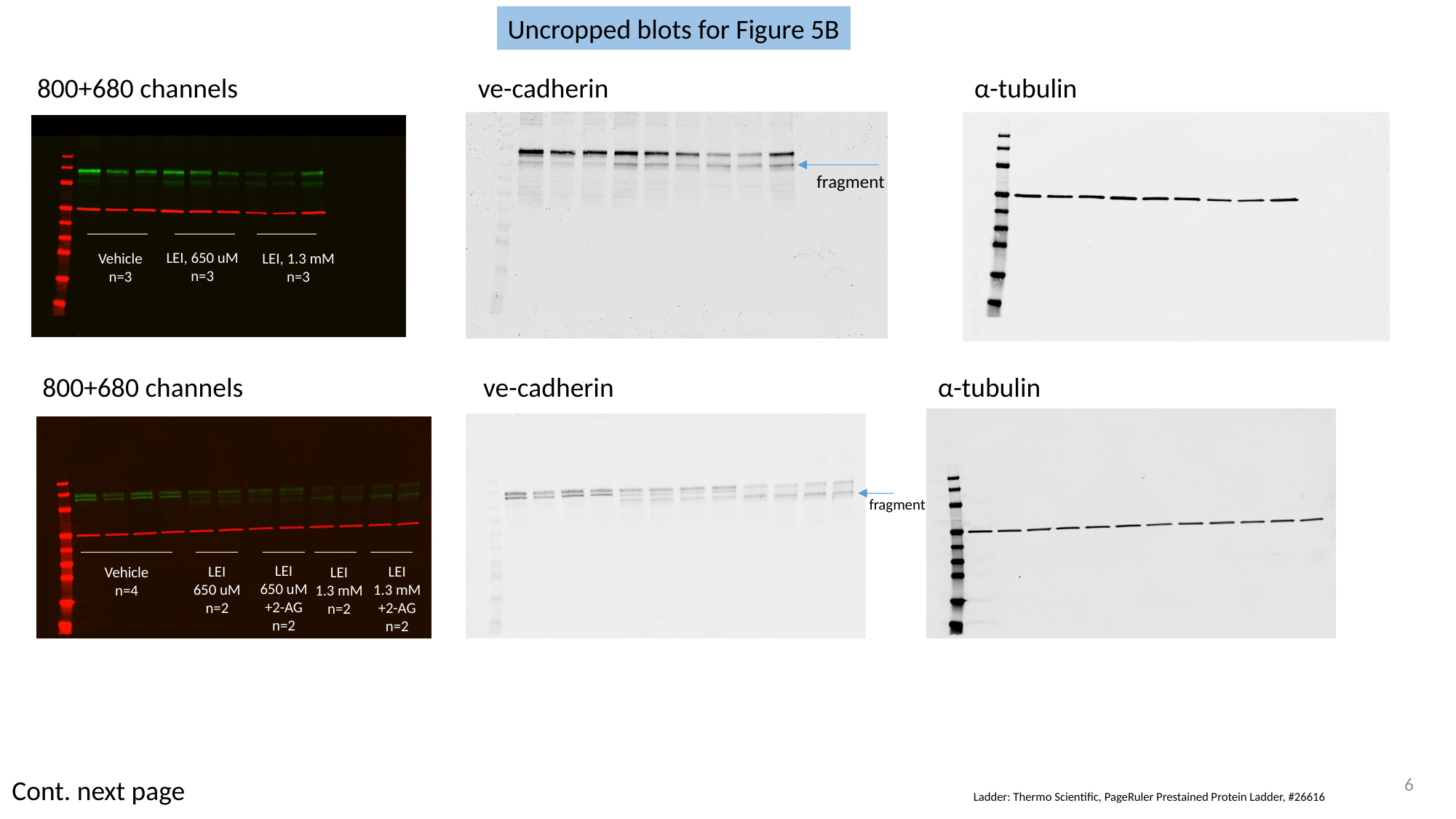

Uncropped blots for Figure 5B
800+680 channels
ve-cadherin
α-tubulin
fragment
LEI, 650 uM
n=3
Vehicle
n=3
LEI, 1.3 mM
n=3
800+680 channels
ve-cadherin
α-tubulin
fragment
LEI
650 uM
+2-AG
n=2
LEI
650 uM
n=2
LEI
1.3 mM
+2-AG
n=2
LEI
1.3 mM
n=2
Vehicle
n=4
6
Cont. next page
Ladder: Thermo Scientific, PageRuler Prestained Protein Ladder, #26616

## Slide 7
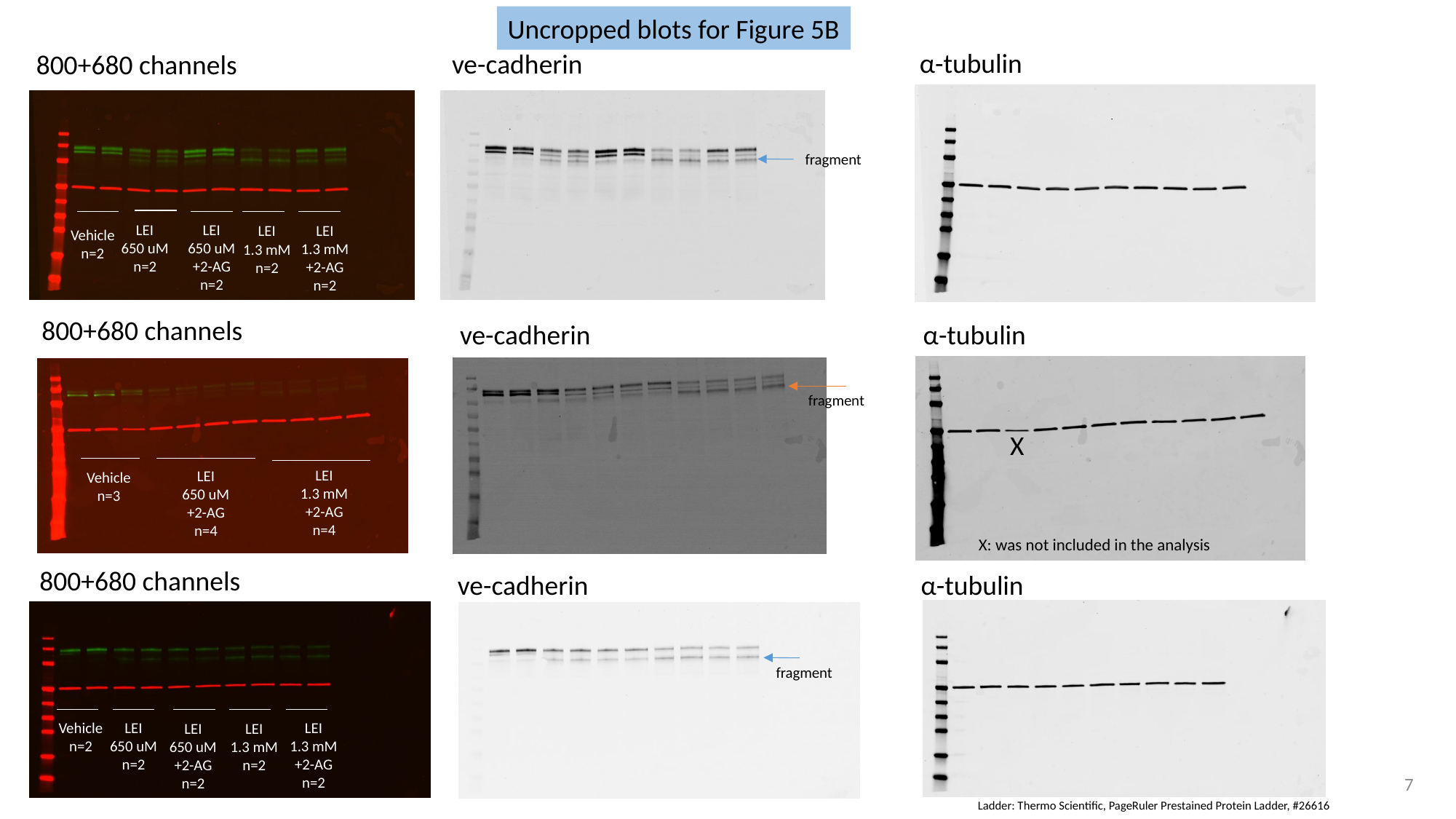

Uncropped blots for Figure 5B
α-tubulin
ve-cadherin
800+680 channels
fragment
LEI
650 uM
+2-AG
n=2
LEI
650 uM
n=2
LEI
1.3 mM
+2-AG
n=2
LEI
1.3 mM
n=2
Vehicle
n=2
800+680 channels
ve-cadherin
α-tubulin
fragment
X
LEI
1.3 mM
+2-AG
n=4
LEI
650 uM
+2-AG
n=4
Vehicle
n=3
X: was not included in the analysis
800+680 channels
ve-cadherin
α-tubulin
fragment
Vehicle
n=2
LEI
650 uM
n=2
LEI
1.3 mM
+2-AG
n=2
LEI
650 uM
+2-AG
n=2
LEI
1.3 mM
n=2
7
Ladder: Thermo Scientific, PageRuler Prestained Protein Ladder, #26616

## Slide 8
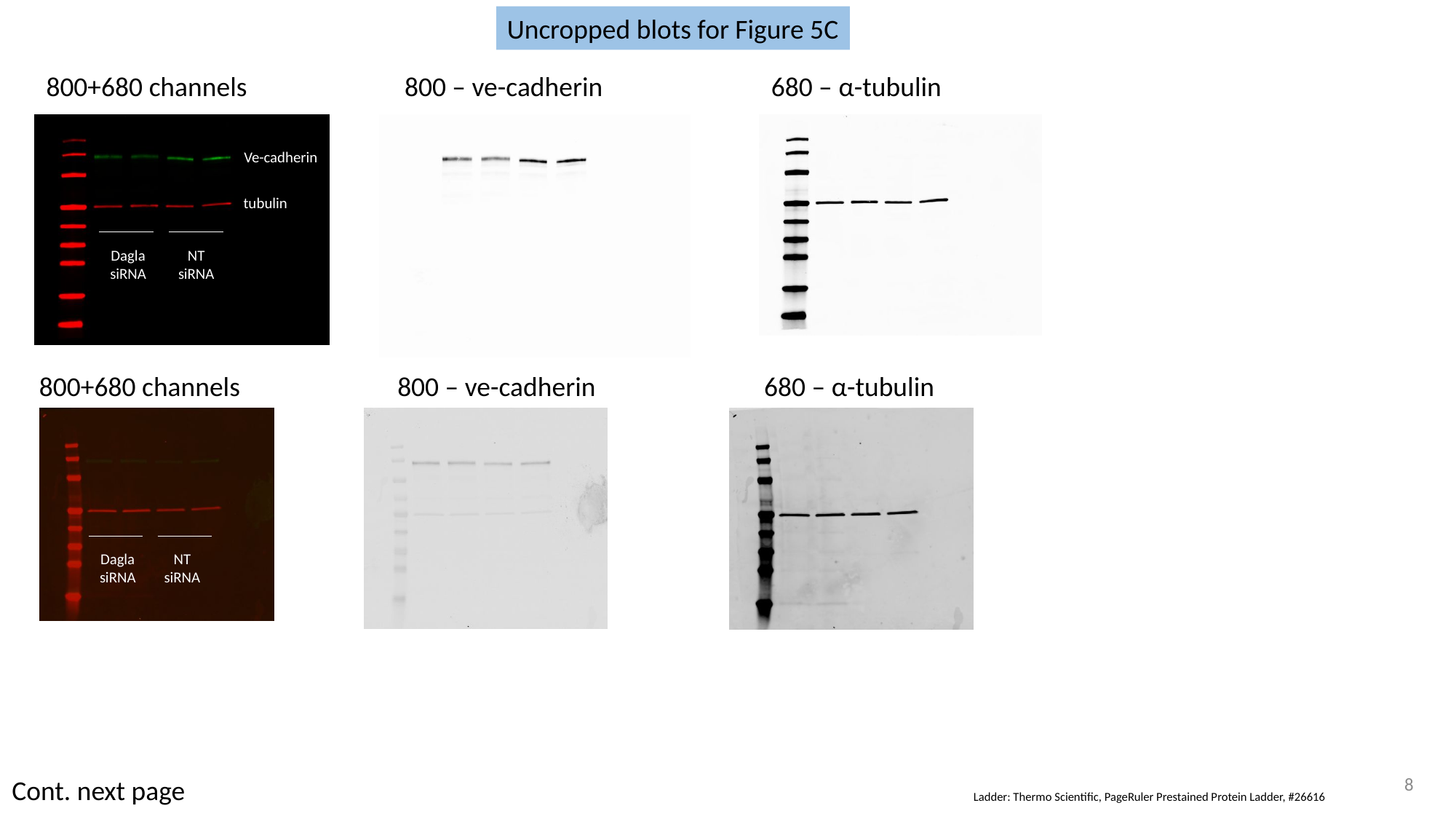

Uncropped blots for Figure 5C
800+680 channels
800 – ve-cadherin
680 – α-tubulin
Ve-cadherin
tubulin
Dagla
siRNA
NT
siRNA
800+680 channels
800 – ve-cadherin
680 – α-tubulin
NT
siRNA
Dagla
siRNA
8
Cont. next page
Ladder: Thermo Scientific, PageRuler Prestained Protein Ladder, #26616

## Slide 9
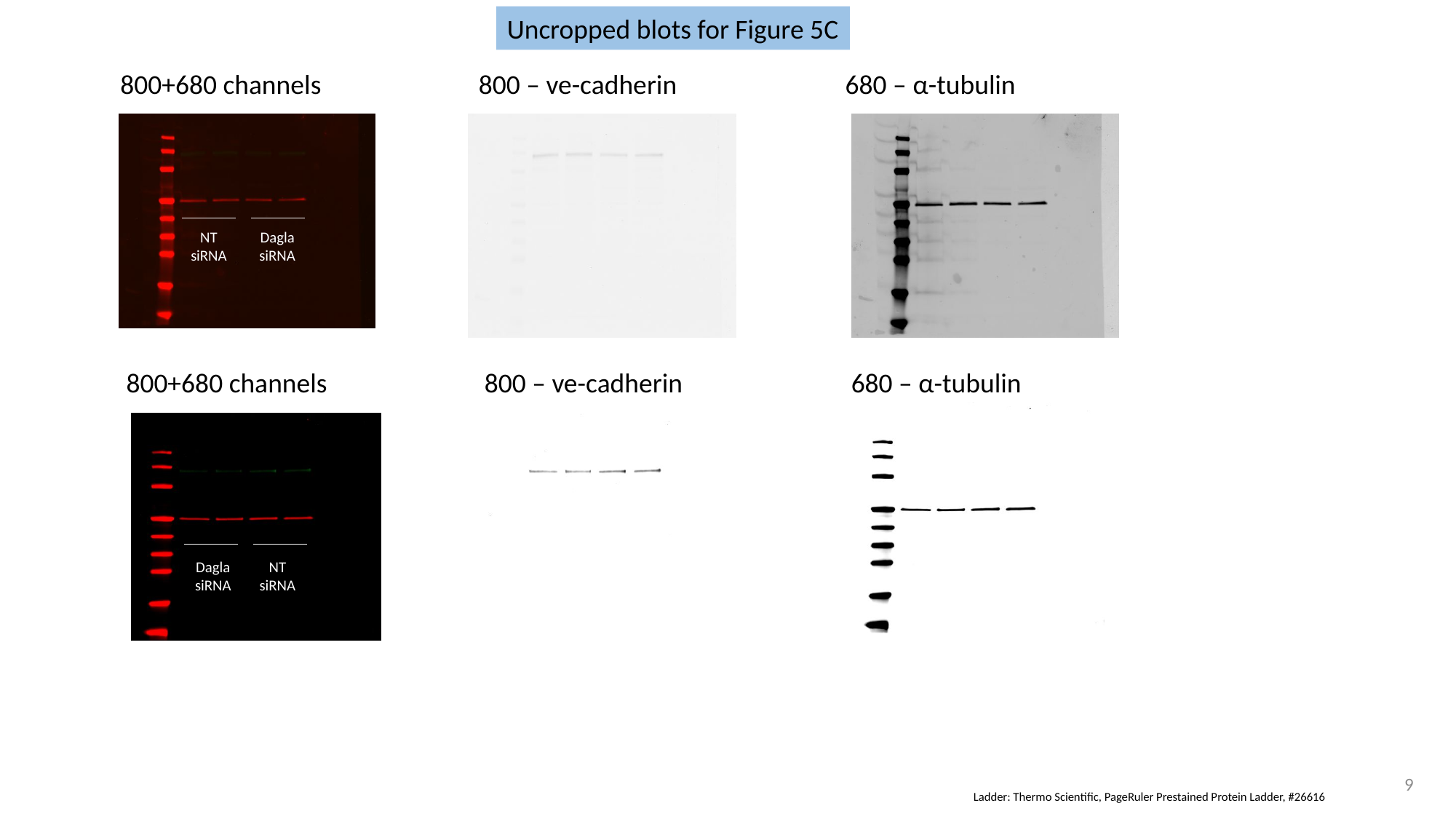

Uncropped blots for Figure 5C
800+680 channels
800 – ve-cadherin
680 – α-tubulin
NT
siRNA
Dagla
siRNA
800+680 channels
800 – ve-cadherin
680 – α-tubulin
NT
siRNA
Dagla
siRNA
9
Ladder: Thermo Scientific, PageRuler Prestained Protein Ladder, #26616

## Slide 10
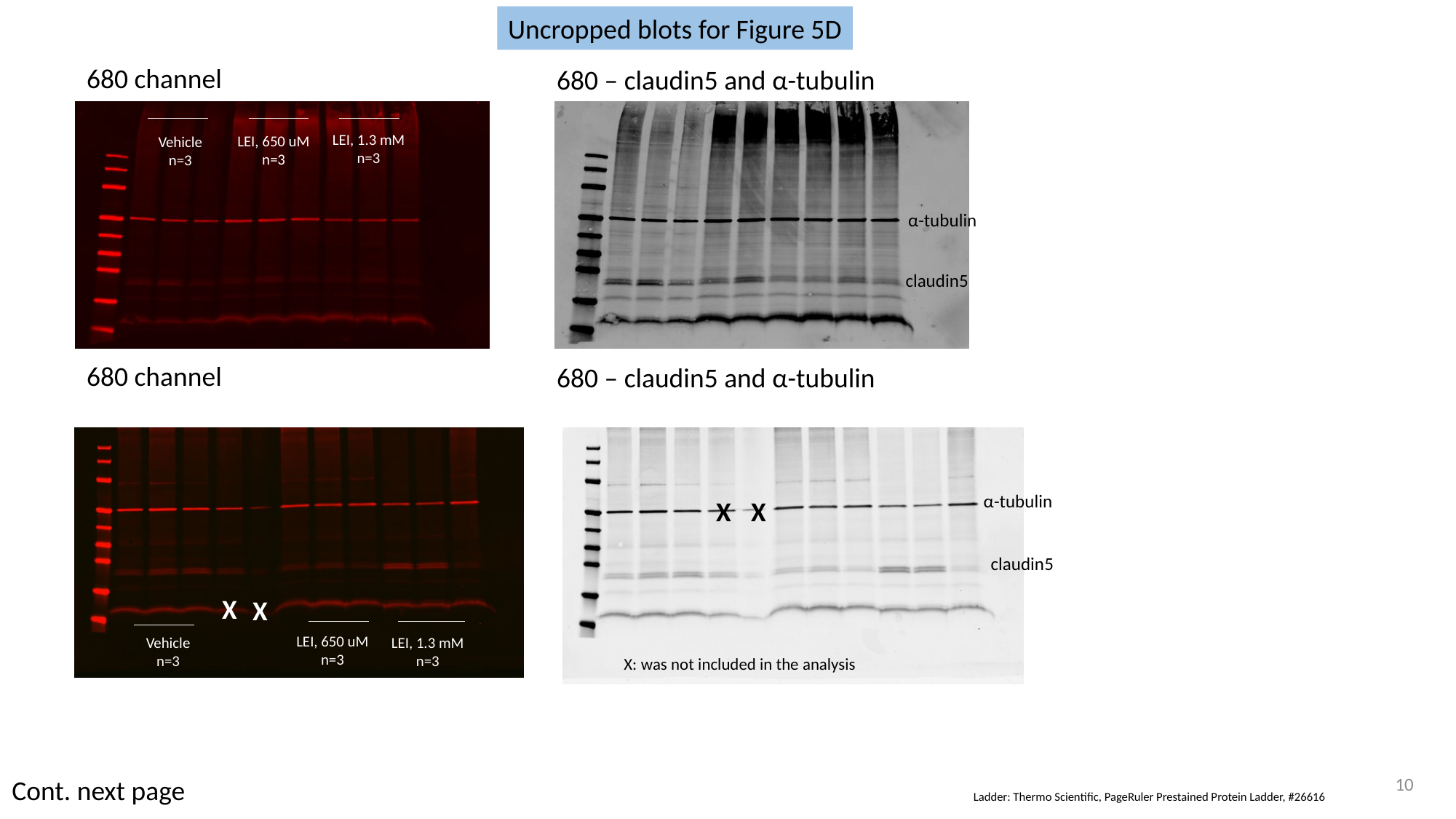

Uncropped blots for Figure 5D
680 channel
680 – claudin5 and α-tubulin
LEI, 1.3 mM
n=3
LEI, 650 uM
n=3
Vehicle
n=3
α-tubulin
claudin5
680 channel
680 – claudin5 and α-tubulin
α-tubulin
X
X
claudin5
X
X
LEI, 650 uM
n=3
Vehicle
n=3
LEI, 1.3 mM
n=3
X: was not included in the analysis
10
Cont. next page
Ladder: Thermo Scientific, PageRuler Prestained Protein Ladder, #26616

## Slide 11
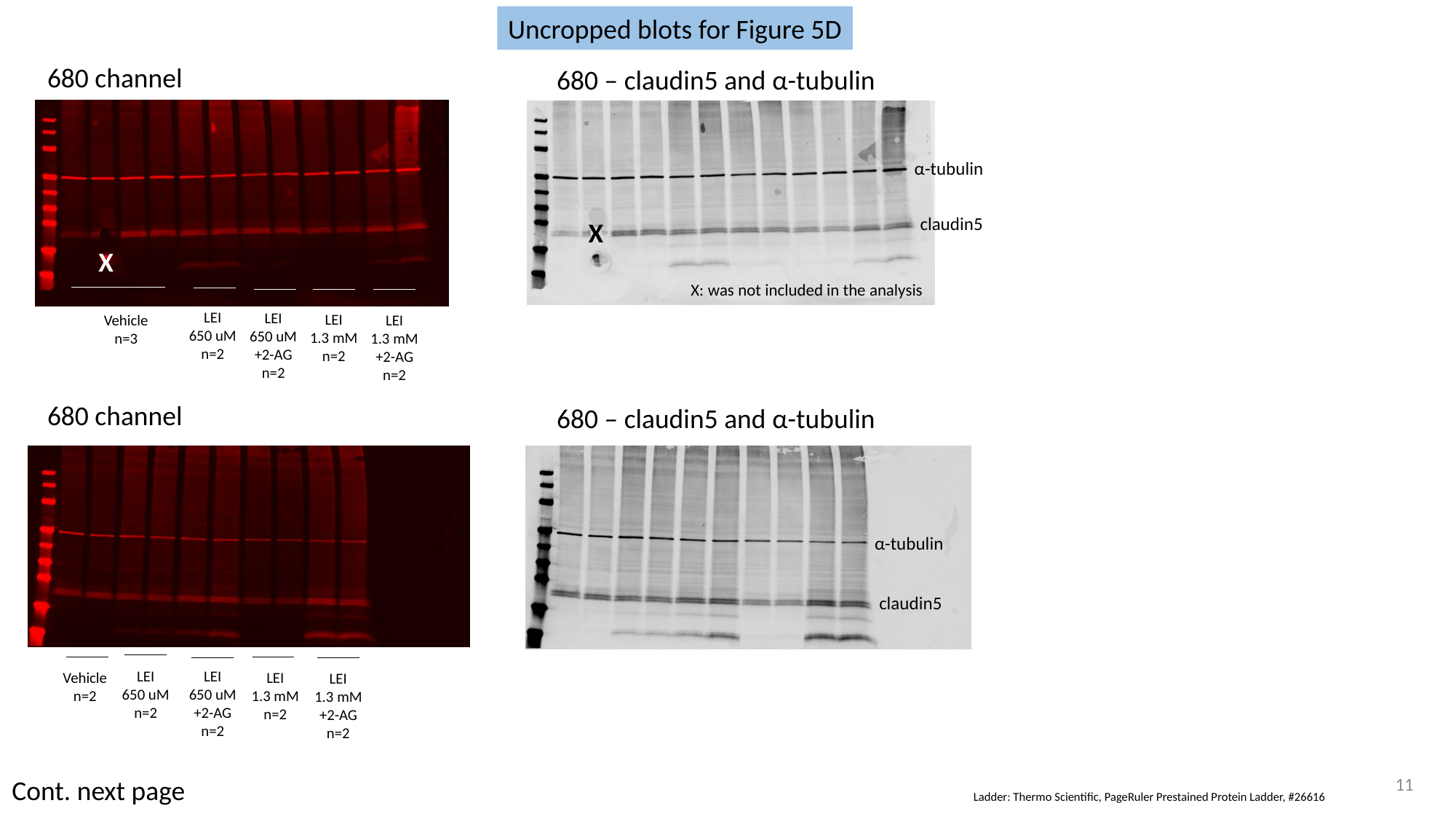

Uncropped blots for Figure 5D
680 channel
680 – claudin5 and α-tubulin
α-tubulin
claudin5
X
X
X: was not included in the analysis
LEI
650 uM
n=2
LEI
650 uM
+2-AG
n=2
LEI
1.3 mM
n=2
Vehicle
n=3
LEI
1.3 mM
+2-AG
n=2
680 channel
680 – claudin5 and α-tubulin
α-tubulin
claudin5
LEI
650 uM
n=2
LEI
650 uM
+2-AG
n=2
Vehicle
n=2
LEI
1.3 mM
n=2
LEI
1.3 mM
+2-AG
n=2
11
Cont. next page
Ladder: Thermo Scientific, PageRuler Prestained Protein Ladder, #26616

## Slide 12
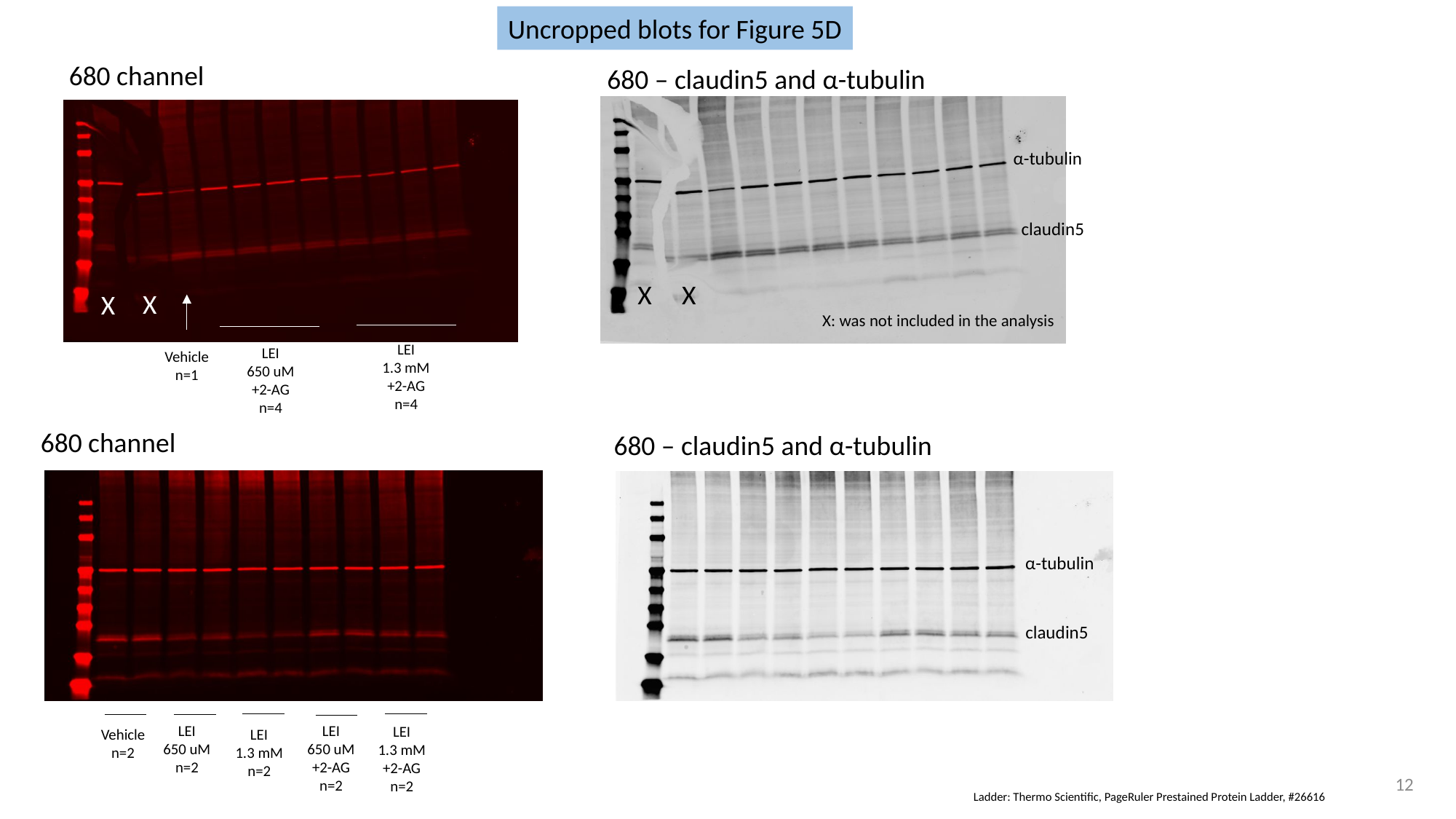

Uncropped blots for Figure 5D
680 channel
680 – claudin5 and α-tubulin
α-tubulin
claudin5
X
X
X
X
X: was not included in the analysis
LEI
1.3 mM
+2-AG
n=4
LEI
650 uM
+2-AG
n=4
Vehicle
n=1
680 channel
680 – claudin5 and α-tubulin
α-tubulin
claudin5
LEI
650 uM
n=2
LEI
650 uM
+2-AG
n=2
LEI
1.3 mM
+2-AG
n=2
Vehicle
n=2
LEI
1.3 mM
n=2
12
Ladder: Thermo Scientific, PageRuler Prestained Protein Ladder, #26616

## Slide 13
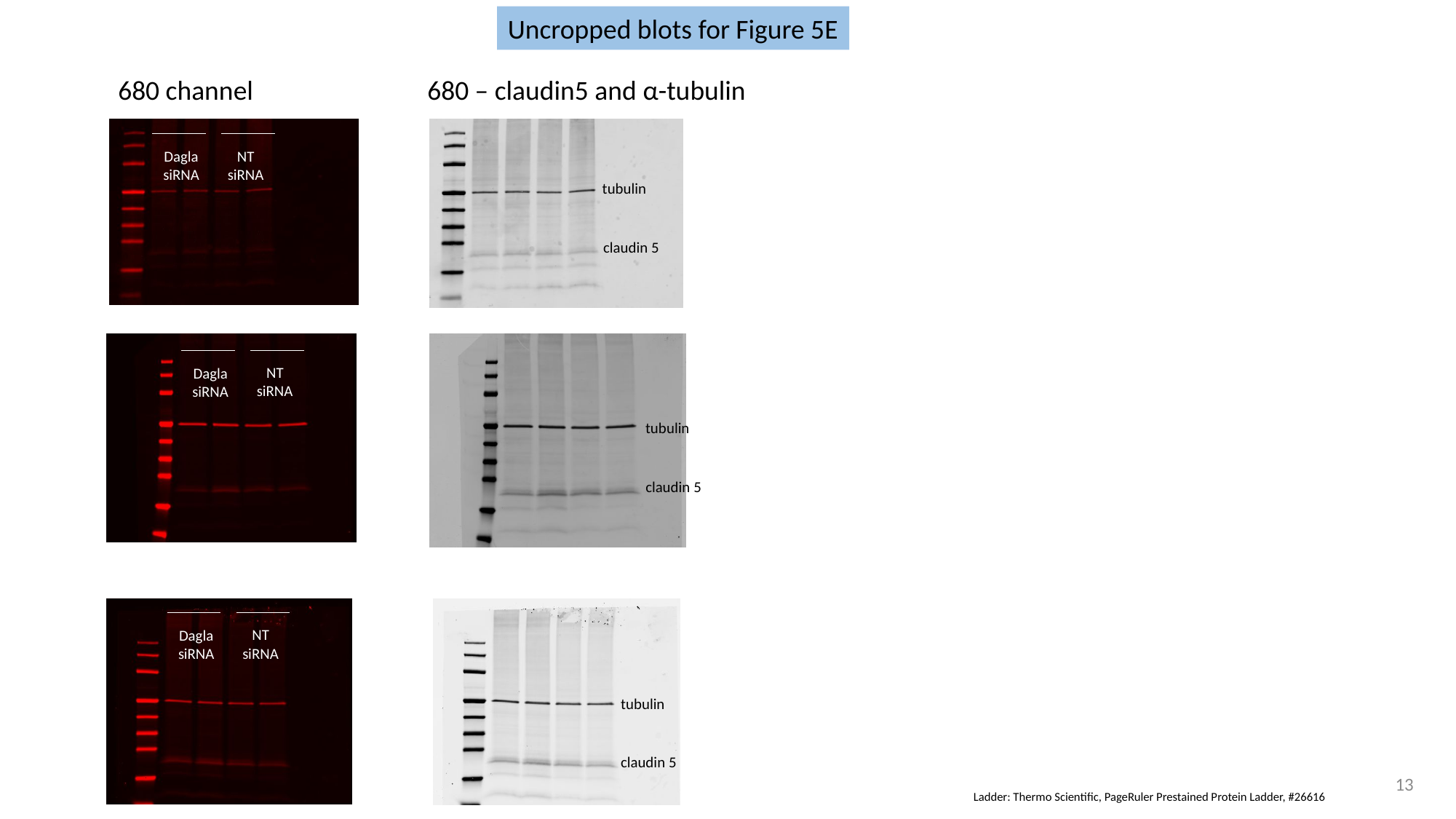

Uncropped blots for Figure 5E
680 channel
680 – claudin5 and α-tubulin
NT
siRNA
Dagla
siRNA
tubulin
claudin 5
NT
siRNA
Dagla
siRNA
tubulin
claudin 5
NT
siRNA
Dagla
siRNA
tubulin
claudin 5
13
Ladder: Thermo Scientific, PageRuler Prestained Protein Ladder, #26616

## Slide 14
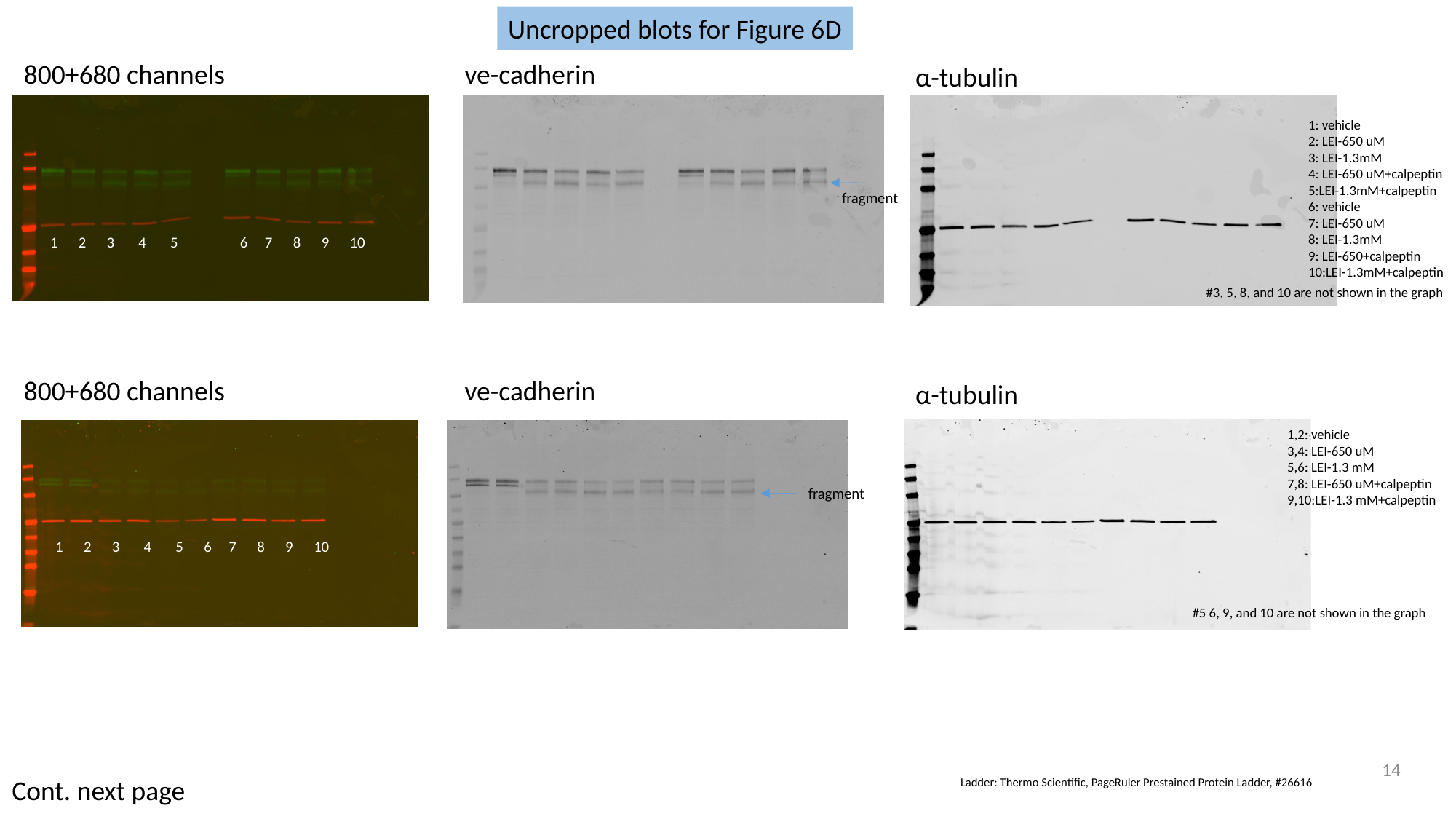

Uncropped blots for Figure 6D
800+680 channels
ve-cadherin
α-tubulin
1: vehicle
2: LEI-650 uM
3: LEI-1.3mM
4: LEI-650 uM+calpeptin
5:LEI-1.3mM+calpeptin
6: vehicle
7: LEI-650 uM
8: LEI-1.3mM
9: LEI-650+calpeptin
10:LEI-1.3mM+calpeptin
fragment
1 2 3 4 5 6 7 8 9 10
#3, 5, 8, and 10 are not shown in the graph
800+680 channels
ve-cadherin
α-tubulin
1,2: vehicle
3,4: LEI-650 uM
5,6: LEI-1.3 mM
7,8: LEI-650 uM+calpeptin
9,10:LEI-1.3 mM+calpeptin
fragment
1 2 3 4 5 6 7 8 9 10
#5 6, 9, and 10 are not shown in the graph
14
Cont. next page
Ladder: Thermo Scientific, PageRuler Prestained Protein Ladder, #26616

## Slide 15
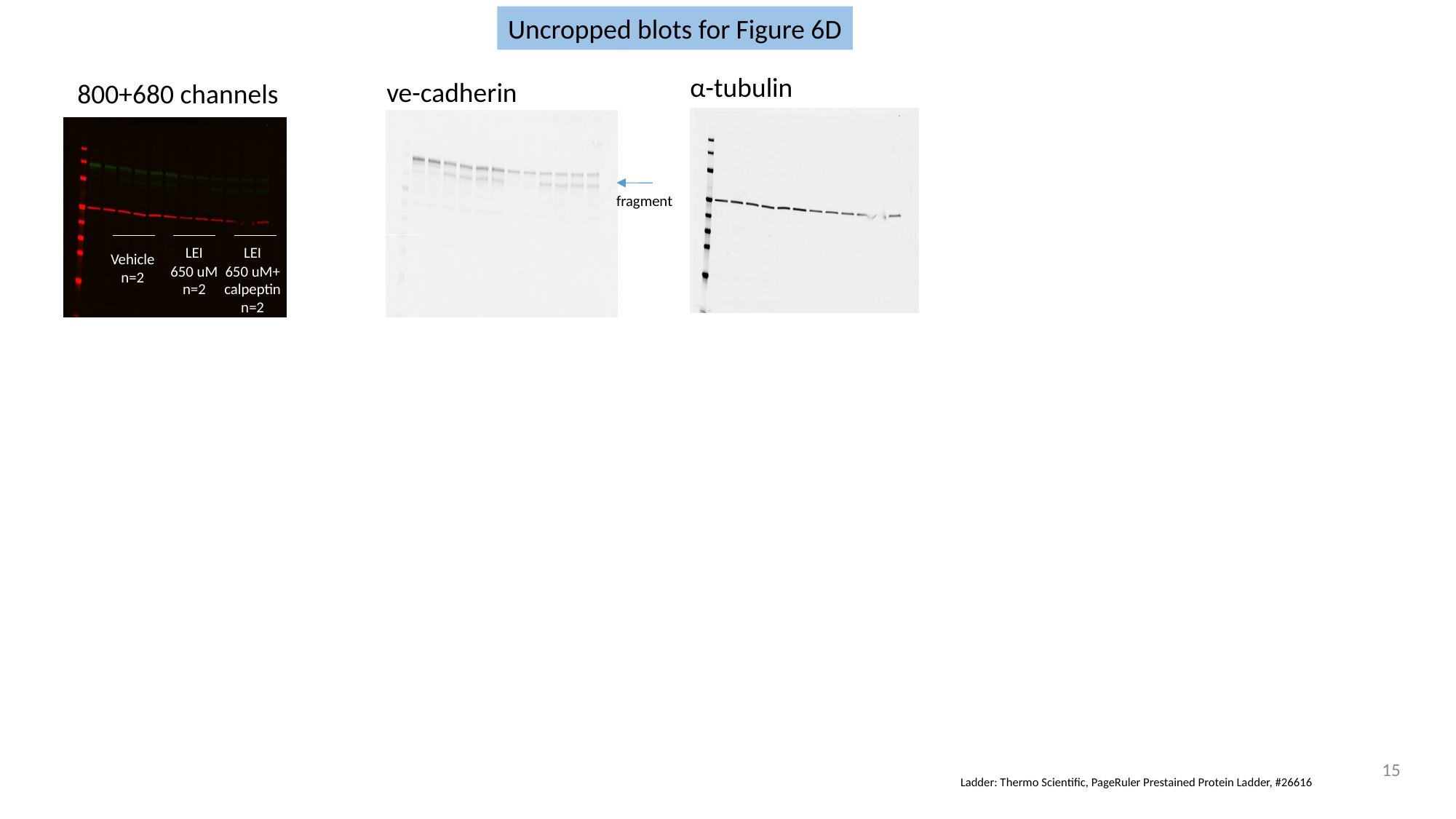

Uncropped blots for Figure 6D
α-tubulin
ve-cadherin
800+680 channels
fragment
LEI
650 uM
n=2
LEI
650 uM+
calpeptin
n=2
Vehicle
n=2
15
Ladder: Thermo Scientific, PageRuler Prestained Protein Ladder, #26616

## Slide 16
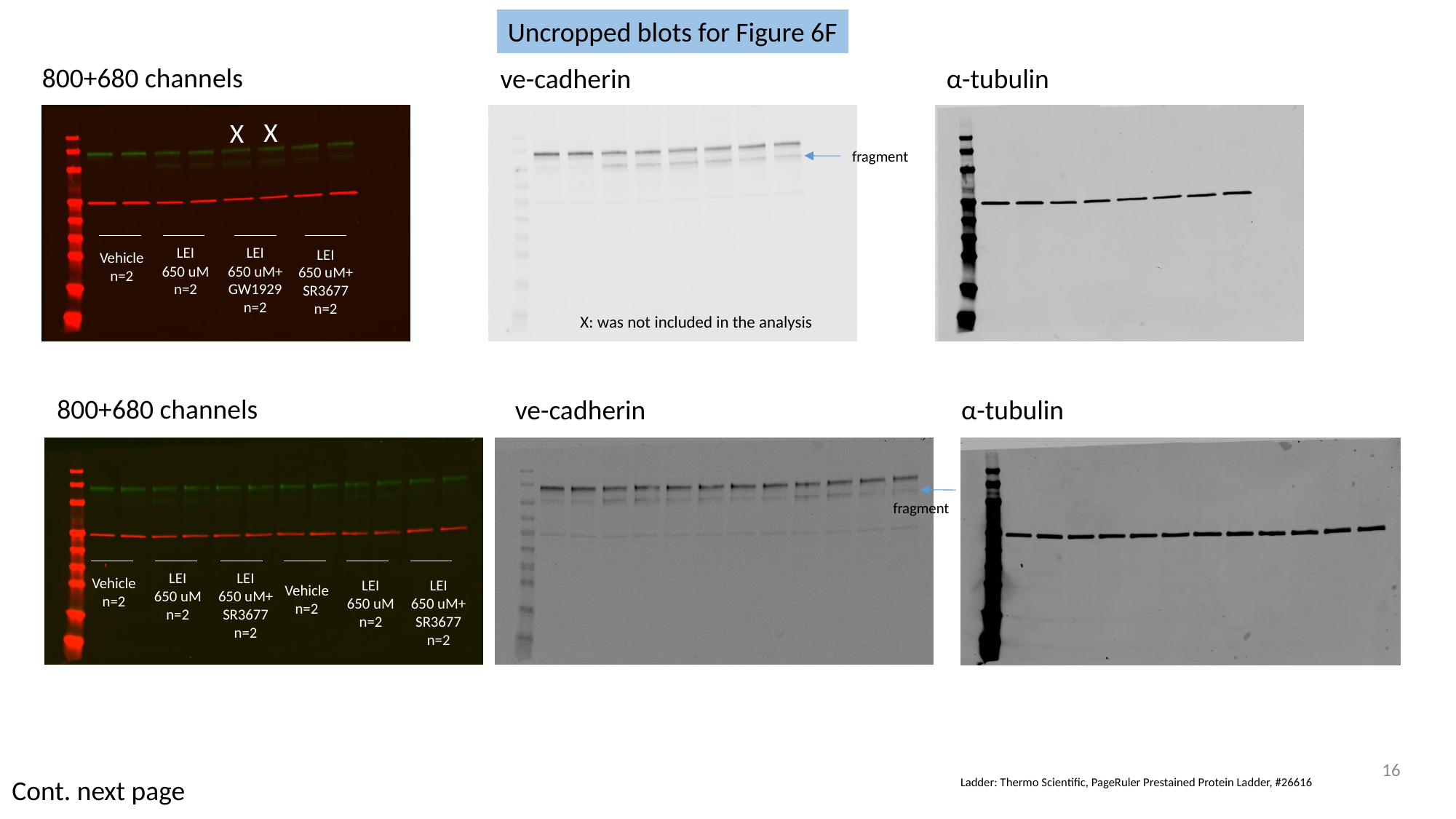

Uncropped blots for Figure 6F
800+680 channels
ve-cadherin
α-tubulin
X
X
fragment
LEI
650 uM
n=2
LEI
650 uM+
GW1929
n=2
LEI
650 uM+
SR3677
n=2
Vehicle
n=2
X: was not included in the analysis
800+680 channels
ve-cadherin
α-tubulin
fragment
LEI
650 uM
n=2
LEI
650 uM+
SR3677
n=2
Vehicle
n=2
LEI
650 uM
n=2
LEI
650 uM+
SR3677
n=2
Vehicle
n=2
16
Cont. next page
Ladder: Thermo Scientific, PageRuler Prestained Protein Ladder, #26616

## Slide 17
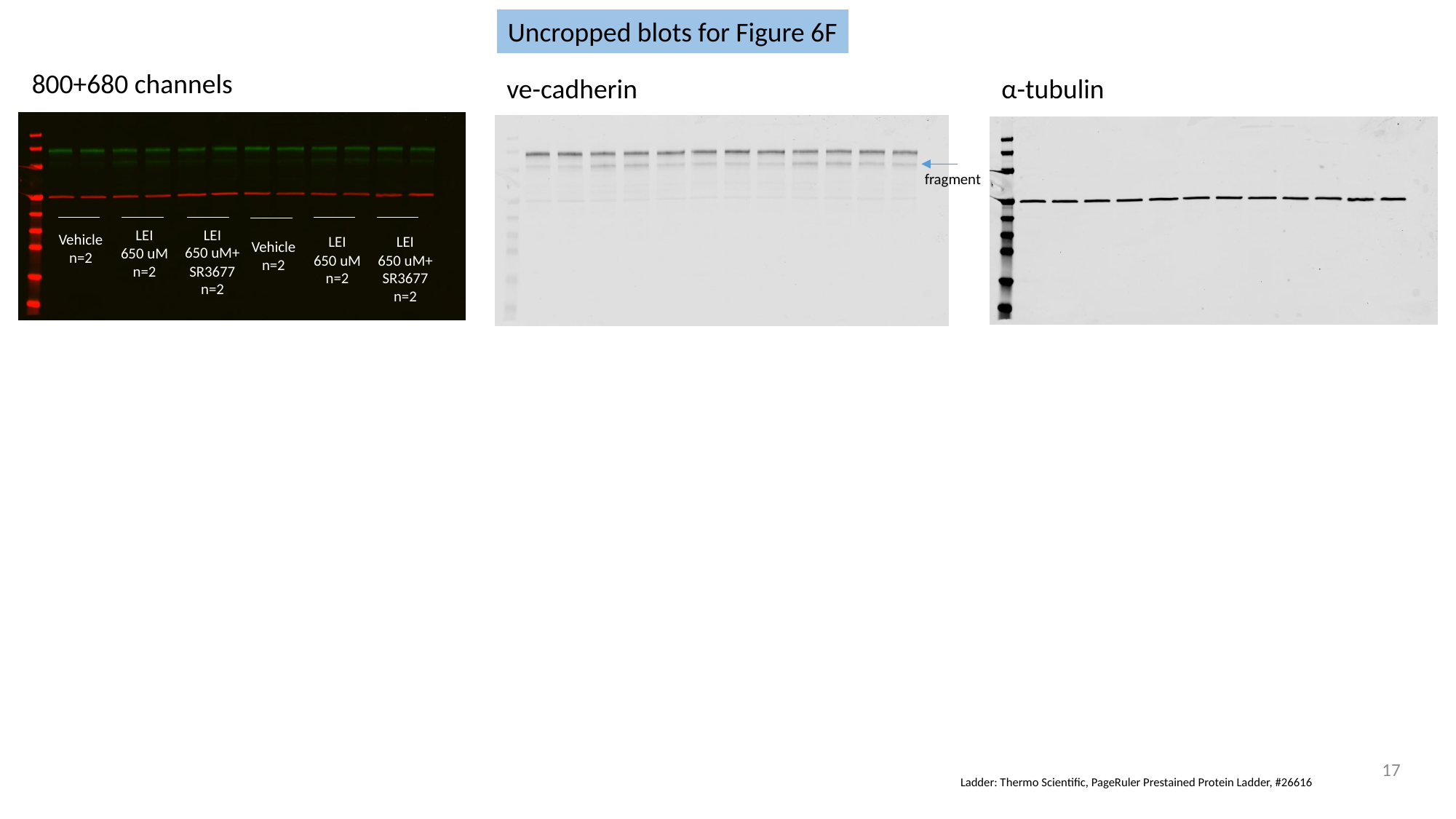

Uncropped blots for Figure 6F
800+680 channels
ve-cadherin
α-tubulin
fragment
LEI
650 uM
n=2
LEI
650 uM+
SR3677
n=2
Vehicle
n=2
LEI
650 uM
n=2
LEI
650 uM+
SR3677
n=2
Vehicle
n=2
17
Ladder: Thermo Scientific, PageRuler Prestained Protein Ladder, #26616

## Slide 18
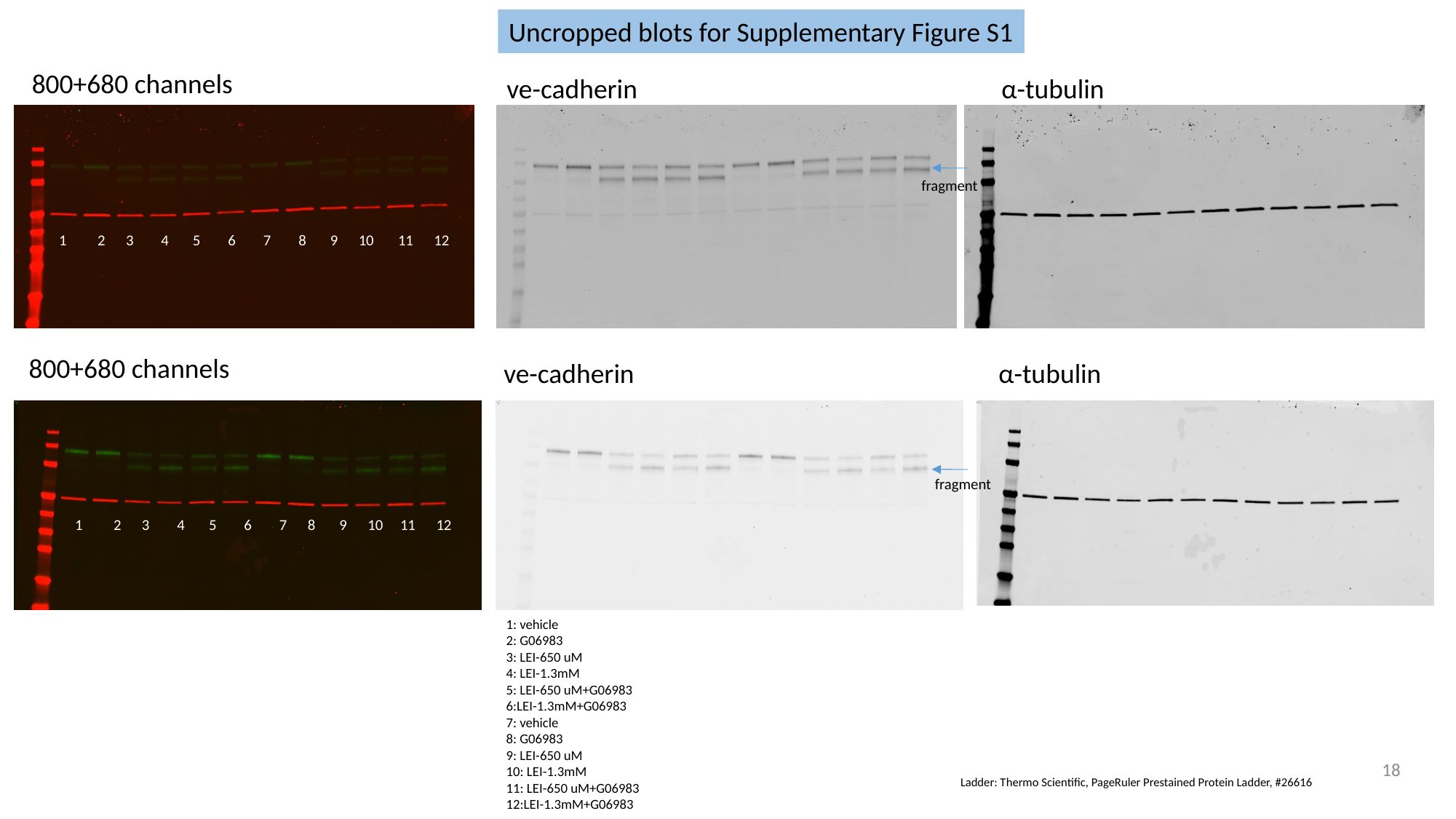

Uncropped blots for Supplementary Figure S1
800+680 channels
ve-cadherin
α-tubulin
fragment
1 2 3 4 5 6 7 8 9 10 11 12
800+680 channels
ve-cadherin
α-tubulin
fragment
1 2 3 4 5 6 7 8 9 10 11 12
1: vehicle
2: G06983
3: LEI-650 uM
4: LEI-1.3mM
5: LEI-650 uM+G06983
6:LEI-1.3mM+G06983
7: vehicle
8: G06983
9: LEI-650 uM
10: LEI-1.3mM
11: LEI-650 uM+G06983
12:LEI-1.3mM+G06983
18
Ladder: Thermo Scientific, PageRuler Prestained Protein Ladder, #26616

## Slide 19
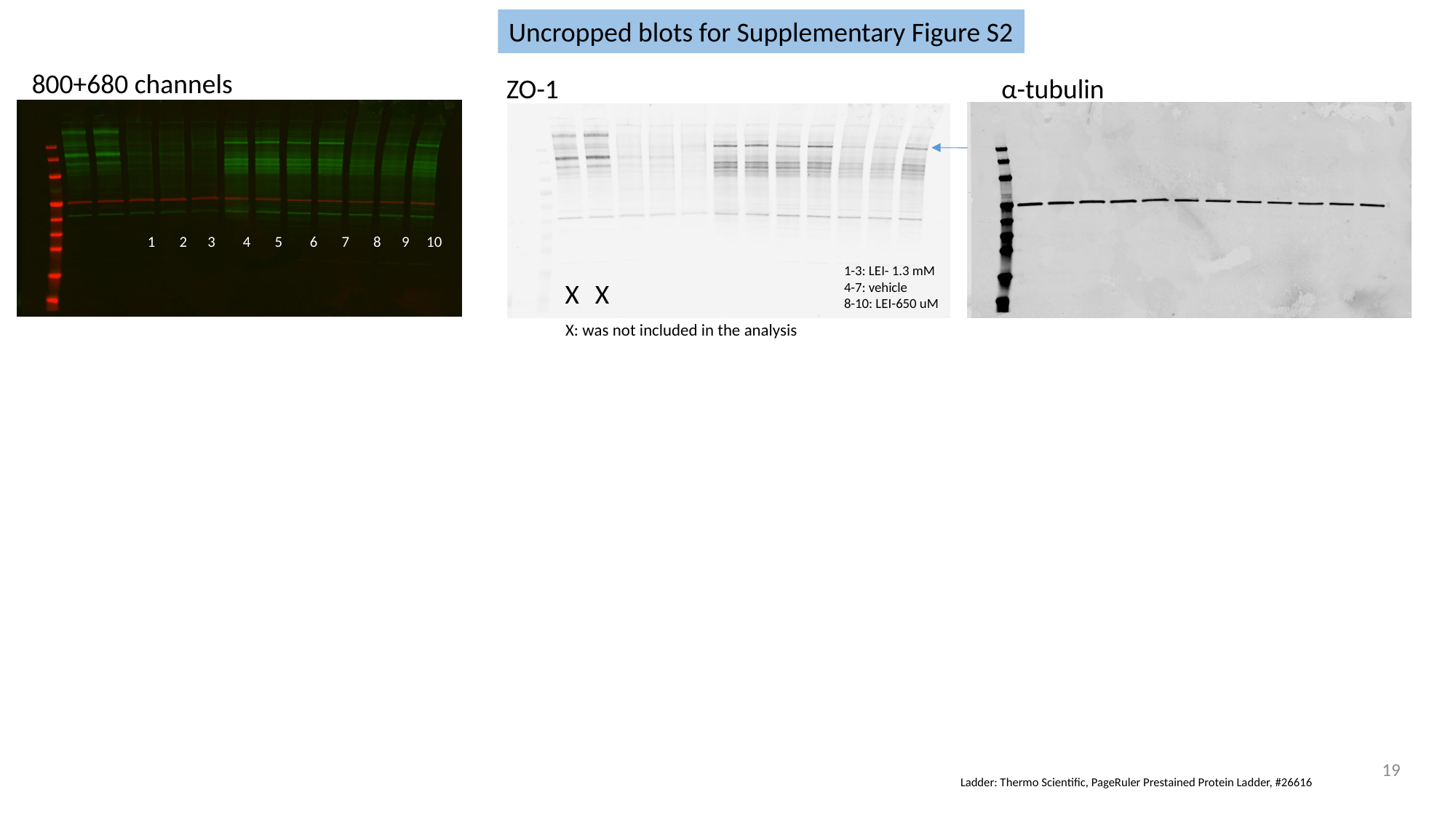

Uncropped blots for Supplementary Figure S2
800+680 channels
ZO-1
α-tubulin
1 2 3 4 5 6 7 8 9 10
1-3: LEI- 1.3 mM
4-7: vehicle
8-10: LEI-650 uM
X
X
X: was not included in the analysis
1 2 3 4 5 6 7 8 9 10 11 12
19
Ladder: Thermo Scientific, PageRuler Prestained Protein Ladder, #26616
